# Obstetric Scar Endometriosis: Retrospective Study on 19 Cases and Review of the Literature

**DOI:** 10.1155/2014/417042

**Published:** 2014-09-18

**Authors:** Mustafa Kaplanoglu, Dilek Kaya Kaplanoğlu, Ceren Dincer Ata, Selim Buyukkurt

**Affiliations:** ^1^Department of Obstetrics and Gynecology, School of Medicine, Adiyaman University, Adiyaman, Turkey; ^2^IMC Private Hospital, Obstetrics and Gynecology, Mersin, Turkey; ^3^School of Medicine, Cukurova University, Adana, Turkey

## Abstract

Endometriosis is defined as the presence of functioning endometrial tissue outside the uterine cavity. This disease is one of the most common gynecologic disorders in reproductive age women. It generally occurs in pelvic cavity. But extrapelvic location has been defined (such as extremities, central nervous system, lungs, pleurae, liver, umbilicus, pericardium, urinary tract, intestines, and surgical scar tissue). Scar endometriosis is a rare disease and defined as presence of endometriotic lesions on the abdominal (such as cesarean section and hysterectomy) or vaginal (episiotomy) excision line. It is difficult to diagnose due to the extreme variability in presentation. The symptoms are nonspecific, typically involving pain, swelling at the incision site at the time of menstruation. Excision and histopathologic examination are necessary for diagnosis. We present a case series of obstetric scar endometriosis and review of the literature.

## 1. Introduction

Endometriosis is defined as finding the functional endometrial layer outside the uterine cavity. It is one of the most common gynecological disorders in the reproductive age women and its prevalence in the general population varies between 0.7 and 44% [1]. Endometriosis is usually found in the pelvis and especially the ovaries, uterosacral ligaments, and round ligaments. On the other hand, it is observed in extrapelvic sites. But it is a rare condition. It affects between 0.03 and 1.7% of reproductive age women. The most common implantation sites are bladder, gastrointestinal tract, lungs, and on the skin especially after obstetric surgical interventions [2]. Surgical scar endometriosis is a rare condition. The main cause of surgical scar endometriosis is obstetric and gynecologic operations (such as in the perineum following vaginal delivery with episiotomy and in abdominal surgery scar areas following hysterectomy and cesarean section). Its clinical diagnosis is difficult and confused with abscess, hematoma, suture granuloma, desmoid tumor, sarcoma, and metastatic malignancy. Several theories have been defined in etiology of scar endometriosis. But most accepted theory is direct implantation of the endometrial tissue in scars during the surgical procedure [3]. The careful anamnesis and physical examination are essential for diagnosis. The knowledge of the clinical background of the disease is essential for diagnosis of scar endometriosis. The evaluation of ultrasound scan is essential in some cases. The definite treatment of extrapelvic endometriosis is by surgery and the diagnosis can be made by histopathological examination of the material. The aim of this surgery is prevention of recurrence of lesions.

The retrospective evaluation and treatment of scar endometriosis cases with obstetric causes that were prediagnosed with clinical imaging methods and verified histopathologically have been presented in this study.

## 2. Material and Method

Nineteen patients who presented to our clinic with symptoms of pain and swelling in the obstetric surgical scar area between January 2008 and May 2013 were evaluated. Physical findings at presentation, age, previous obstetric operative time, duration of symptoms, localization of lesions, the size of the mass, treatment method, and histopathological results of the patients were registered (Table 1). The postsurgical diagnosis was performed using histopathological analysis, in which the criterion was the presence of endometrial glands and/or stromal cells in the connective tissue that was analyzed. All the patients underwent surgical removal of the lesions with a safety margin. The diagnosis was confirmed by the pathological anatomical examination. This research project was approved by the Research Ethics Committee of Adiyaman University School of Medicine. All patients were then evaluated with these data.

## 3. Description

The time of last obstetric surgical intervention: it is defined as the time between diagnosis and last obstetric surgical intervention.

## 4. Result

**Figure 1:**
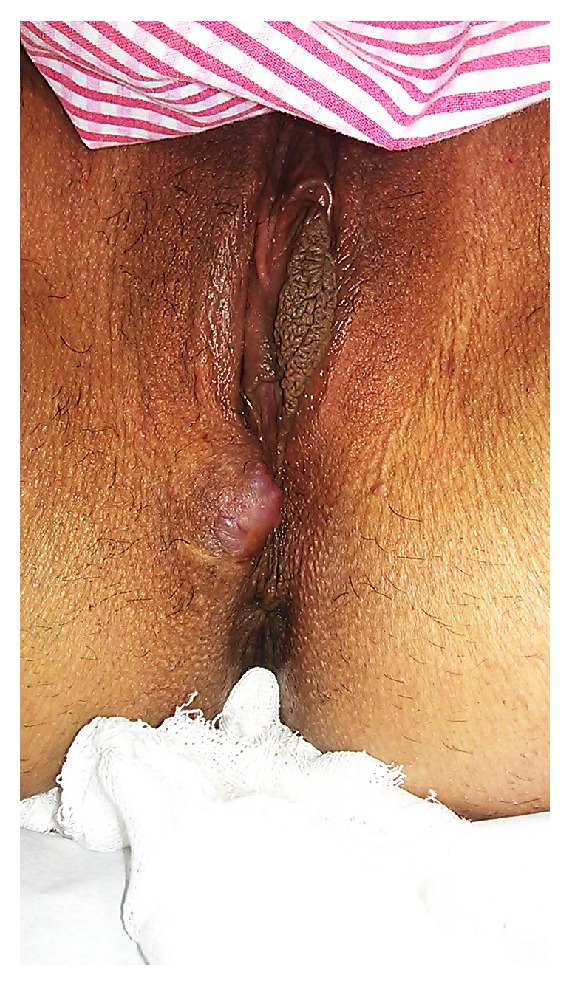
Endometriotic lesion is apparent in episiotomy area.

A total of 19 patients who had presented to our clinic with symptoms of swelling and pain in 13 caesarean section and 6 episiotomy scars between January 2008 and May 2013 were evaluated (Figure 1). All patients received a preliminary diagnosis of obstetric incision scar endometriosis preoperatively due to their history and examination characteristics. Ultrasonography was requested in 6 patients and MRI analysis in 5 patients.

The mean age was 29.1 ± 5.4, with range from 21 to 40 years, and the mean number of pregnancies was 2.3 ± 0.5, with range from 1 to 6 pregnancies. All of patients were described as abdominal surgery (cesarean section) and vaginal surgery (episiotomy). Thirteen (68.4%) patients were cesarean section, and six (31.6%) patients were vaginal delivery with episiotomy.

The time between diagnosis and last obstetric surgical intervention was 45.4 ± 32.5 months, with range from 12 to 108 months in evaluation of all patients. This period was 16.8 ± 13.8 months, with range from 0,5 to 48 months for cesarean section patients and 56.0 ± 36.1 months, with range from 12 to 108 months for vaginal delivery with episiotomy patients. The mean duration of the symptoms, defined as the time between symptoms and the first visit, was 19.7 ± 15.1 months, with range from 0,5 to 48 months in evaluation of all patients. This result was 26 ± 17.1 (0,5–48) months in vaginal delivery with episiotomy patients and was 16.8 ± 13.8 (0,5–48) months in cesarean section patients. The mean asymptomatic period, defined as the time interval between surgical intervention and onset of symptoms, was 23.2 ± 20.7 months.

**Figure 2:**
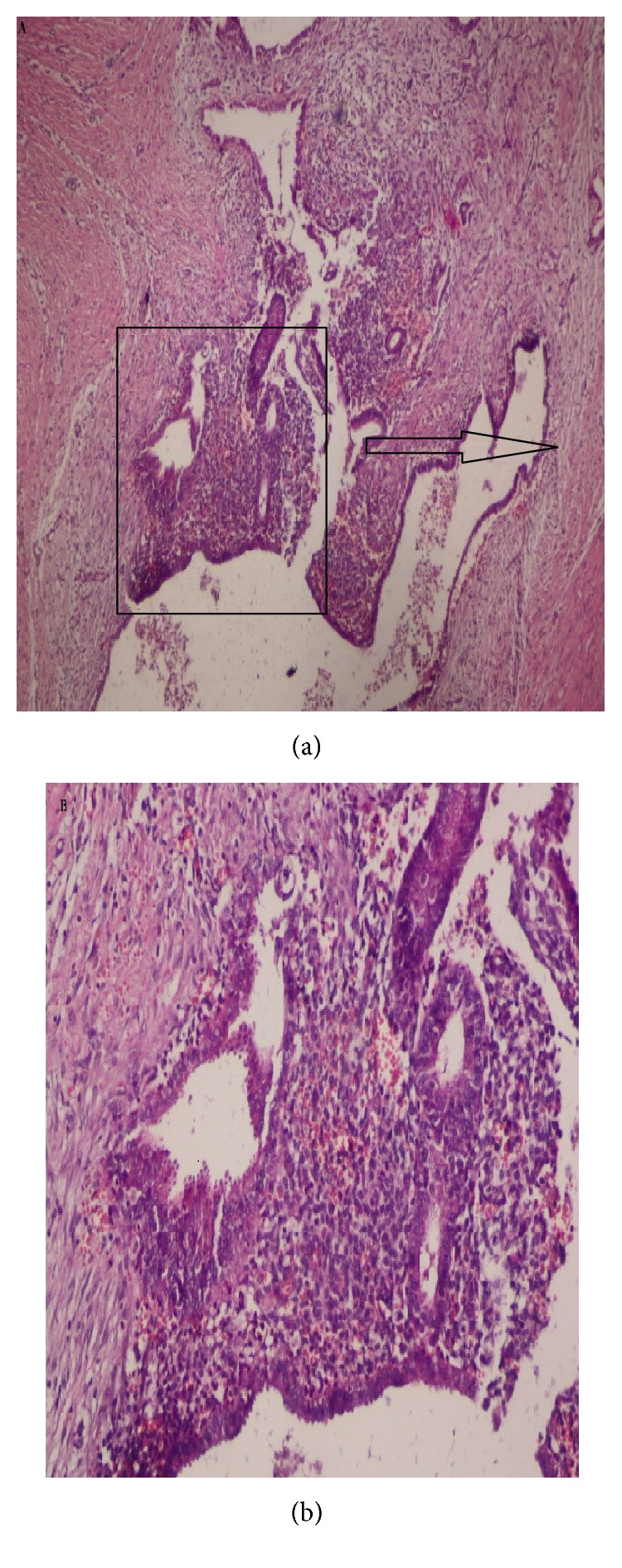
(a) Histopathologic appearance of endometriotic lesions 4 × 1. (b) Histopathologic appearance of endometriotic lesions 10 × 2.

Tumoral tissue was surgically excised with a 1 cm surgical margin of safety in all patients. A fascial defect that developed in two patients after surgical excision was repaired with prolene suture material by us. The final diagnosis was reported as endometriosis on histopathological examination (Figure 2). The mean follow-up period after surgery was 24 (6–35) months and no recurrence was observed. None of patients received the additional treatment modalities because of endometriosis like symptoms were not described in the gynecologic history of patients.

A total 43934 births with 14252 cesarean sections and 29682 normal births took place at our clinic in the specified period. The incidence of obstetric scar location endometriosis was 0.09% for cesarean section and 0.04% for vaginal delivery in our clinic. As a result, cesarean section is one of the most important risk factors for obstetric scar endometriosis in our data (OR: 4,51, Cl: 1,72–11,87). This result may be according to surgical method and amount of endometriotic tissue in scar area.

## 5. Discussion

Endometriosis is defined as finding endometrial gland and stroma outside the uterus. Although this placement is usually in the pelvis, it may be outside the pelvis and in tissues such as the lung, ureter, brain, and obstetric scars [4–7]. There are many theories on endometriosis development but the subject is still controversial. However, direct mechanical implantation of endometrial tissue seems to be the most appropriate theory for the explanation of the scar endometriosis. Development of scar endometriosis requires implantation of the cells in the relevant area following abdominal or vaginal procedures, protection from the immune response, and hormonal stimulus for cell growth. On the other hand, predisposing factors such as smoking and alcohol consumption should not be ignored [8].

It is difficult to determine the precise incidence of obstetric scar endometriosis due to the wide range of obstetric scar locations, follow-up durations, and the clinical presentation. While the incidence of endometriosis was as high as 10% among women of reproductive age, the incidence of abdominal wall endometriosis after cesarean section was 0.03–1.7% and the incidence of episiotomy region endometriosis after normal birth was 0.06–0.7% in studies with a limited number of cases [4, 9]. The results are consistent with our findings. On the other hand, these types of lesions can be observed as skin lesions after procedures such as laparoscopy and amniocentesis.

An important issue besides the symptoms caused by the lesions themselves such as swelling and pain is malignant transformation of scar endometriosis. The incidence of malignant transformation in scar endometriosis is 0.3–1%. Functional complications (such as defecation and sexual problems) according to endometriosis surgery are commonly observed in extensive surgery. The aim of this surgery is prevention of recurrence of lesions. The diagnosis of malign transformation of scar endometriosis is generally postsurgical histopathologically. The most commonly seen malignancy is clear cell carcinoma. Definite histopathological diagnosis of every excised tissue is therefore important [10]. The possibility of malignant transformation of the lesion should be considered in cases that recur after surgery. This rare but important condition should be considered in all patients and monitored carefully during patient follow-up.

History and physical examination are important in detecting scar endometriosis and the most common findings are swelling, pain, and rarely bleeding in the lesion area. Similar results can be caused by many disorders such as hematoma, neuroma, hernia, granuloma, and neoplasia, which should also be considered in the differential diagnosis. Scar endometriosis can be diagnosed with high accuracy with careful physical examination and history. Scar endometriosis was considered as the preliminary diagnosis in all our cases after obtaining the medical history and was confirmed histologically. Menstruation-related pain and swelling in the anamnesis should be considered to be pathognomonic for scar endometriosis. However, a definite diagnosis can be made by histopathological examination of the lesion.

Although the basis for preliminary diagnosis is the medical history and physical examination, imaging methods such as ultrasound, MRI, CT, and fine-needle aspiration biopsy are helpful in the diagnosis. Ultrasonography is the most commonly used imaging method. Sonographic features are not specific. Irregular border, internal heterogeneous echotexture, and increased vascularity may be present. Occasionally, cystic changes may be present. A hypoechoic heterogeneous lesion with internal echoes indicates scar endometriosis. If there is a suspicion of deep invasion, MRI provides useful information [11]. However, imaging methods only aid the diagnosis and are not usually required. Fine-needle aspiration biopsy is an important method to confirm the histological diagnosis. Macrophages loaded with endometrial glands, stroma, and hemosiderin on histological examination are diagnostic. However, implantation of cells in other tissues and organ perforation in hernia cases are major complications in case of probable malignancy. Local excision of the mass provides an opportunity for both diagnosis and treatment, limiting the importance of fine-needle aspiration biopsy.

Surgical removal of the lesion is necessary for the diagnosis and treatment and the complete excision of the lesion together with approximately 1 cm of healthy tissue is important to prevent local recurrence. After the excision, the repair of defect with synthetic mesh is necessary in patients with an extensive facial defect. It is a rare condition and generally the repair is made by plastic surgeon. But we did not detect extensive facial defect according to endometriosis in our clinic. Anal sphincter invasion in episiotomy scar endometriosis is a rare condition requiring primary sphincter repair [12]. In this situation, general surgeon was invited an operation.

Many methods have been proposed for the prevention of scar endometriosis. The closure of the visceral and parietal peritoneum during cesarean section and the use of high doses of progesterone during the first 6 months after hysterectomy are the main suggestions [13, 14]. On the other hand, washing the incision area with saline after cesarean section can reduce the risk of developing endometriosis by reducing cell burden.

The patients who were evaluated in our study had presented to our clinic with pain and a mass in the cesarean or episiotomy scar region. The risk factor was considered to be the obstetric interventions. Surgical excision was performed for all lesions with approximately 1 cm of disease-free tissue margin. Defective areas were primarily sutured, and the tissue defect in 2 patients was repaired with polypropylene mesh. All material received after surgery was examined and the histopathological diagnosis of endometriosis was confirmed. No recurrence was observed during postoperative follow-up. Our findings indicate that a 1 cm safety margin is adequate during tumor excisions. However, it should be noted that the surgical technique and the safety margin might vary according to the location of the lesion [15, 16]. The data found in our presented study conforms to the results of many studies in the literature [9].

Scar endometriosis has now become even more important due to the increased number of obstetric surgical interventions. However, the high rate of diagnosis with a simple physical examination and medical history is an important advantage. Prevention of other tissues by endometrial tissue as much as possible during obstetric interventions can be recommended for the prevention of scar endometriosis. Excision of lesions with a margin of safety is important, and we suggest that this margin can be 1 cm. Prospective studies with a large number of patients are needed for progress on the prevention of scar endometriosis.

## Figures and Tables

**Table 1:** Clinical features and treatment of the patient.

Patient	Age	Operation previous (months)	Type of obstetric operation	Time of symptoms (months)	Location of lesion	Size of mass (cm)	Presenting symptoms	Imaging exams	Treatment
1	28	48	Vaginal delivery	30	On the episiotomy scar	4 × 2 × 2	Mass, cyclic pain, swelling	—	Excision
2	29	48	C/S	24	RLS	3 × 3 × 3	Mass, cyclic pain	US, MRI	Excision
3	38	108	Vaginal delivery	48	On the episiotomy scar	3 × 2 × 2	Mass, cyclic pain, swelling	—	Excision
4	31	24	Vaginal delivery	12	On the episiotomy scar	2 × 2 × 2	Mass, cyclic pain	—	Excision
5	21	24	C/S	12	RLS	4 × 4 × 3	Mass, cyclic pain, swelling	US, MRI	Excision
6	25	36	C/S	24	LLS	3 × 3 × 3	Mass, cyclic pain	US	Excision
7	24	24	C/S	12	RLS	4 × 2 × 2	Mass, cyclic pain	—	Excision
8	23	48	C/S	12	LLS	4 × 2 × 2	Mass, cyclic pain	US, MRI	Excision
9	28	12	C/S	6	RLS	2 × 2 × 1	Mass, cyclic pain	—	Excision
10	40	84	Vaginal delivery	30	On the episiotomy scar	2 × 2 × 2	Mass, cyclic pain, swelling	—	Excision
11	34	96	C/S	48	LLS	3 × 3 × 2	Mass, cyclic pain	US, MRI	Excision
12	36	108	C/S	36	RLS	3 × 3 × 3	Mass, cyclic pain, swelling	—	Excision
13	30	36	C/S	24	RLS	2 × 2 × 1	Mass, cyclic pain	—	Excision
14	31	60	Vaginal delivery	36	On the episiotomy scar	3 × 2 × 2	Mass, cyclic pain	—	Excision
15	26	24	C/S	12	RLS	3 × 3 × 3	Mass, cyclic pain	US, MRI	Excision
16	28	12	C/S	6	RLS	4 × 2 × 2	Mass, cyclic pain	—	Excision
17	35	48	C/S	2	LLS	4 × 3 × 2	Mass, cyclic pain	—	Excision
18	23	12	C/S	0.5	LLS	3 × 2 × 2	Mass, cyclic pain	—	Excision
19	24	12	Vaginal delivery	0.5	On the episiotomy scar	2 × 2 × 2	Mass, cyclic pain, swelling	—	Excision

RLS: right lateral side of incision; LLS: left lateral side of incision; US: ultrasonography; MRI: magnetic resonance imaging.
